# A meta-analysis and trial sequential analysis of high intensity focused ultrasound ablation combined with transhepatic arterial chemotherapy and embolization for hepatoma

**DOI:** 10.3389/fonc.2022.1025177

**Published:** 2022-10-27

**Authors:** Liang Zhang, Kuishuai Xu, Xuehui Zhang, Linqian Li, Jing Chong, Ning Yu

**Affiliations:** ^1^ Department of Abdominal Ultrasound, the Affiliated Hospital of Qingdao University, Qingdao, China; ^2^ Department of Sports Medicine, the Affiliated Hospital of Qingdao University, Qingdao, China

**Keywords:** High Intensity Focused Ultrasound Ablation, Transhepatic Arterial Chemotherapy And Embolization, Trial Sequential Analyses, meta-analysis, hepatoma

## Abstract

**Objective:**

The efficacy of High Intensity Focused Ultrasound Ablation(HIFU) combined with Transhepatic Arterial Chemotherapy And Embolization(TACE) versus TACE alone in the treatment of hepatoma was evaluated by meta-analysis and trial sequential analyses(TSA).

**Methods:**

Pubmed, Cochrane, Embase, Web of Science, Scoups and CNKI, CQVIP, Wanfang Data(China National Knowledge Infrastructure) databases were searched from database construction to April 2022, and randomized controlled trials were included. Revman and Stata software were used for meta-analysis of tumor changes, survival rate, laboratory indicators and adverse reactions in the included studies, and TSA0.9 was used for sequential analysis. Grade Pro was also used to evaluate the included indicators.

**Results:**

Twelve studies were included with a sample size of 1025 cases. Meta-analysis showed that the tumor response rate in the combined treatment group was 1.54 times higher than that in TACE alone (OR: 2.54; 95%CI:1.81-3.57) and the 6-month to 5-year survival rate was 1-4 times higher, with statistically significant differences (P<0.05). Subgroup analysis showed that country, pathological type and study type were the sources of heterogeneity. Egger results showed that there was no publication bias (95%CI: -1.333, 3.552; Ppublication=0.276), and the sensitivity analysis results were reliable. TSA results suggest that there may be false positive results, which need to be further confirmed by more studies. Grade evaluation results indicated that the quality of evidence for response rate and one-year survival was low.

**Conclusion:**

HIFU combined with TACE has better efficacy in the treatment of hepatoma, which is worthy of promotion. However, there may be false positive results in this study, which needs to be further verified by more extensive and more tests.

## Introduction

At present, the death rate of hepatoma has ranked the third in the world (1, 2), next only to gastric cancer and lung cancer. The traditional treatment for liver cancer is laparotomy and laparoscopic resection. However, due to the occult nature of the occurrence of the disease, most patients have developed to the advanced stage when they are detected, thus missing the best treatment period. With the progress of medical treatment, there are more treatment options for liver cancer with different tumor sizes, metastasis and vascular invasion, including transplantation, chemotherapy, radiotherapy, interventional therapy (3), gene therapy (4), biological therapy, etc.

According to current studies and reports, transcatheter arterial chemoembolization has a significant therapeutic effect on hepatoma (5, 6). TACE works by injecting chemotherapeutic drugs or embolization agents to make tumors die due to lack of blood supply, thus achieving the purpose of treatment. Some studies have predicted that the median overall survival rate of TACE alone for intermediate hepatocellular carcinoma is 19.9 months (7). However, due to the high rate of distant metastasis and recurrence of lesions after TACE, combined treatment will be selected for patients clinically. Masatoshi et al. (8) showed that TACE combined with targeted drugs in the treatment of unresectable hepatocellular carcinoma improved progression-free survival compared with TACE alone. Duan et al. (7) studied TACE combined with radiofronous ablation (RFA) in the treatment of hepatocellular carcinoma, and the results suggested that the median progression-free survival was 10 months and the median overall survival was extended by 26 months. The above indicated that TACE combination therapy achieved better efficacy for liver cancer.

The principle of HIFU focuses ultrasonic energy on the tumor, brings energy around the target through heat accumulation, and ablates the tumor through spot placement. HIFU has the advantage of being non-invasive and reproducible. Due to the timely transfer of heat from flowing blood, lesions near blood vessels can avoid vascular damage (9, 10). Especially for patients with advanced cirrhosis, the non-invasive nature of HIFU has more significant advantages (11). A Chinese study found that HIFU can be used as an alternative for unresectable liver cancer (12). The purpose of this study was to compare the efficacy of HIFU combined with TACE versus TACE alone in the treatment of unresectable liver cancer.

## Materials and methods

### Literature retrieval

**Figure 1:**
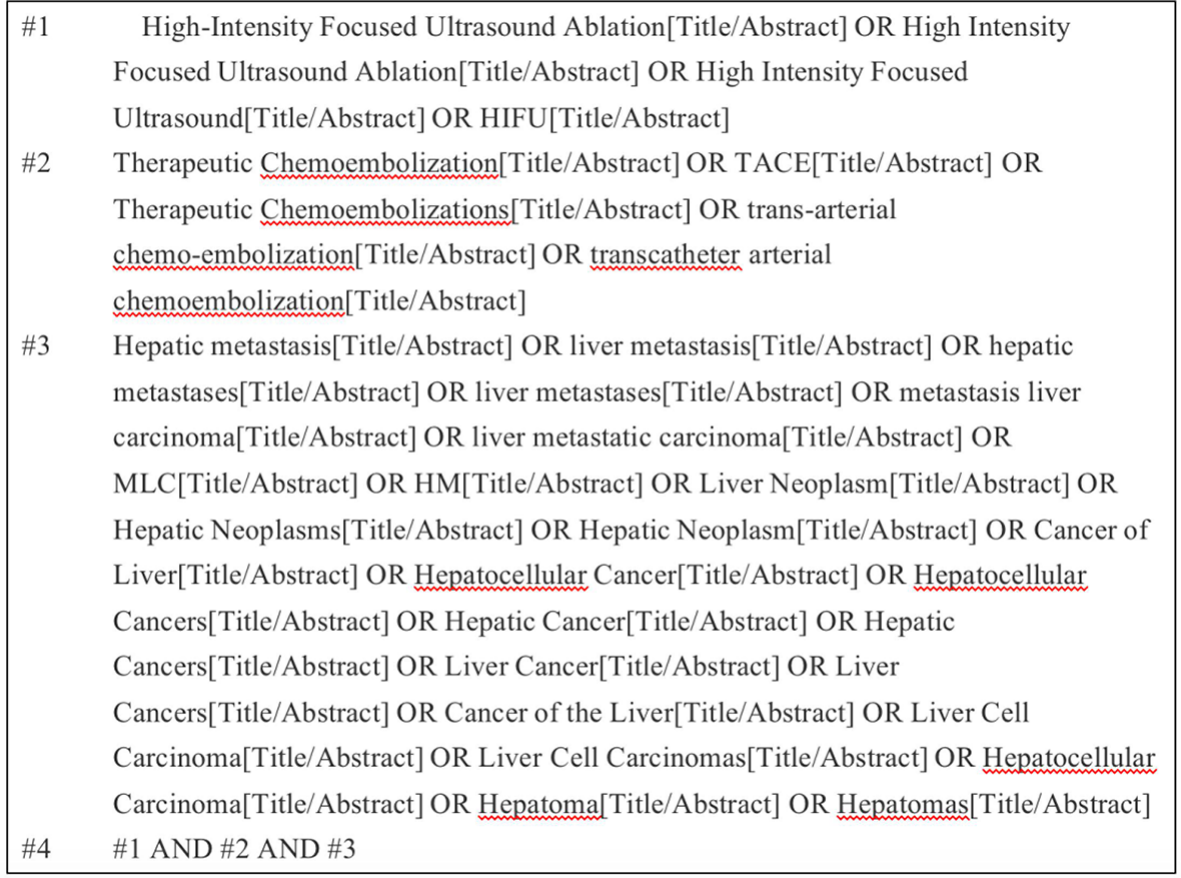
Literature retrieval strategy.

Target databases were searched, including Pubmed, Cochrane, Embase, Web of Science, Scoups, and CNKI. Search for articles published before April 2022 by relevant subject headings. Taking Pubmed as an example, the retrieval strategy is presented (**Figure 1**).

### Inclusion criteria

A. The experimental group was treated with HIFU+TACE, and the control group was treated with TACE alone; B. Outcome indicators include survival analysis results or efficacy analysis results, adverse reactions after surgery, and laboratory test indicators; C. The study type is randomized controlled trial; D. Chinese and English only; E. The subjects of the study were patients with liver cancer (confirmed by corresponding pathological results).

### Exclusion criteria

A. Inconsistent article types, including review, meta-analysis, conference abstract, case report and animal experiment; B. Repeated articles; C. lack of endings; D. Surgical protocols combined with other sequential therapies; E. The control group was treated with HIFU alone.

### Literature quality and risk assessment

According to the above criteria, two researchers independently screened the literature, and summarized the screened literature and extracted the data. Cochrane checklist (1): Random sequence generation; (2) Allocation concealment; (3) Building of participants and personnel; (4) Building of outcome assessment; (5) Incomplete outcome data; (6) Selective reporting; (7) Other bias. Literature quality was evaluated by the above seven indicators. When the opinions of two researchers are inconsistent, the evaluation results of the third researcher are referred to.

### Statistical method

#### Statistics section of Revman

The screened articles were included in Revman v5.4. OR (Odds Ratio, OR) was used to calculate the bivariate data included in the statistics, WMD (Weighted mean difference) was used to calculate the continuous variables, and 95% confidence intervals (CI) were used for interval estimation. Heterogeneity test was performed for preoperative and postoperative changes in laboratory parameters and postoperative adverse reactions in the experimental and control groups. When P ≥ 0.1 and I^2^ ≤ 50%, the results suggested no or low heterogeneity among the included studies, and fixed effects model (FE) was used; on the contrary, when P < 0.1 and I^2^ > 50%, the results suggested large heterogeneity among the included studies, and random effects model (RE) was used.

#### Statistics section of Stata SE15

The effective tumor response of the included articles was statistically analyzed. The overall response rate is considered as an event, and the overall response rate = CR (complete response) + PR (partial response) + SD (stable disease). PD (progressive disease) was set to be event-free. The literature was tested for heterogeneity, and the analysis methods were the same as above. Continue to analyze the heterogeneity literature and explore the source of heterogeneity. After calculating the OR value, a labbe diagram was drawn to quantitatively evaluate the heterogeneity of the articles. After entering two commands (“gen logor = log (ES)”,”gen selogor = selogES”), the articles were plotted with funnel plot and Egger plot, and the articles were assessed for publication bias as well as sensitivity analysis (analyzing the effect of each article on the study).

#### Statistics section of trial sequential analysis

In this study, TSA0.9 software developed by Copenhagen Trial Unit was used on the basis of java program. The type I error probability α = 5% and power is set to 80%. The required information size (RIS) was estimated based on the calculated tumor response efficiency, one-year survival, and I^2^, respectively. Observe if the Z curve crossed the TSA boundary value and if the desired information value was met.

### Grade evaluation

Grade Profiler 3.6 software was used to evaluate the evidence quality of outcome indicators. The results included one-year survival rate and response rate. Among them, the factors that may reduce the quality of RCT evidence include: risk of bias, inconsistency, indirection, imprecision, publication bias; Factors that may improve the quality of evidence in RCTS include large effect sizes, dose-effect relationships, and negative bias. After the evaluation, the recommendation strength was interpreted according to the quality of evidence and the results of meta-analysis.

## Results

### Retrieval results

**Figure 2:**
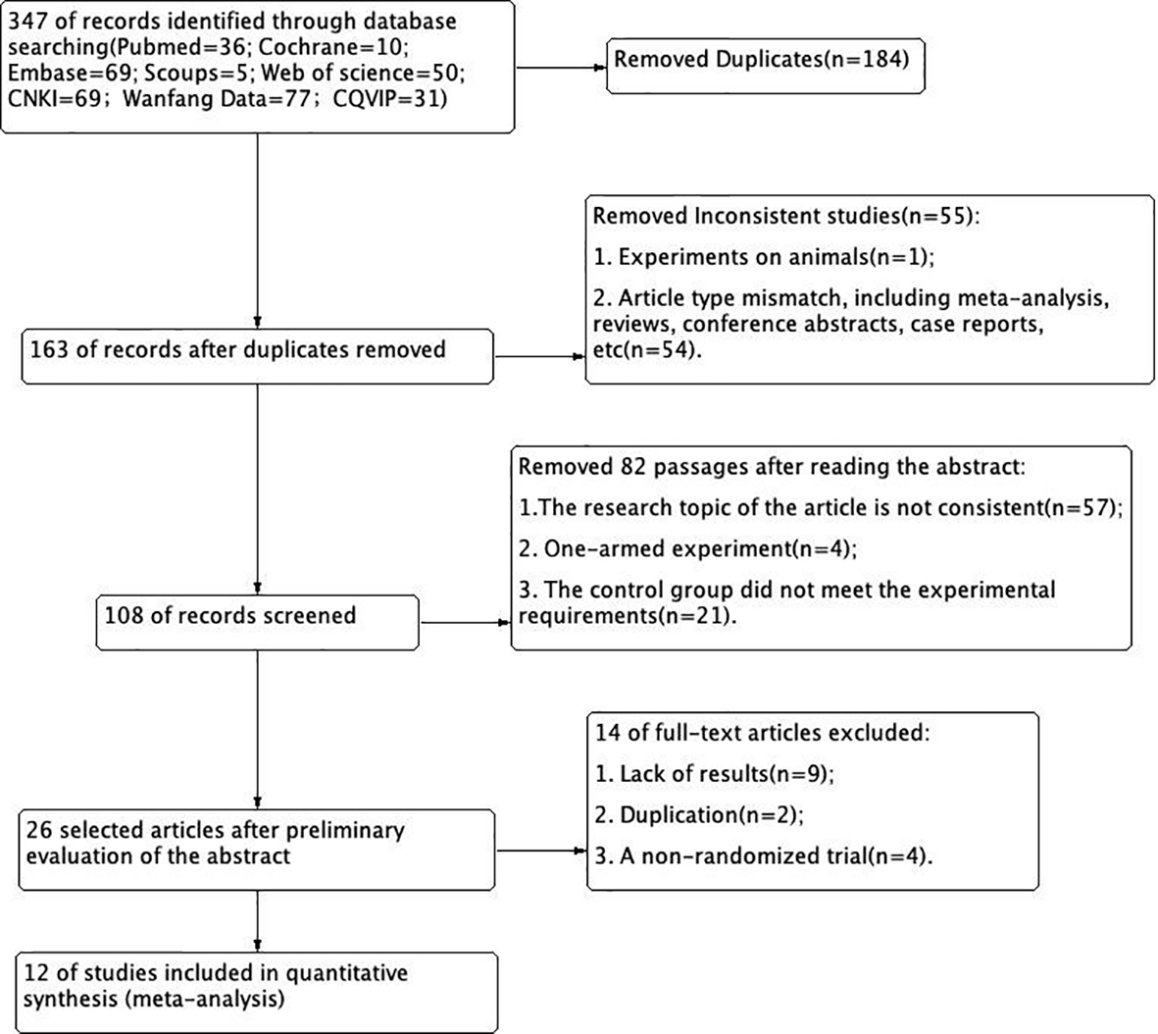
Selection process of included literature.

According to the retrieval conditions, a total of 347 literatures were retrieved. A total of 12 eligible articles (13–24) were selected for meta-analysis (**Figure 2**).

### Basic data and article quality assessment

**Figure 3:**
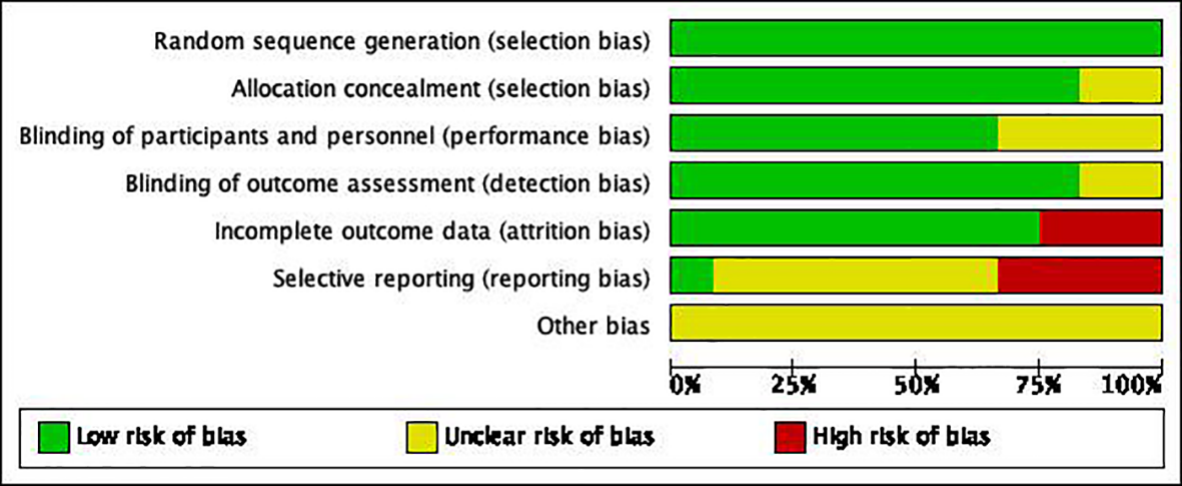
The evaluation results of article quality were included.

**Figure 4:**
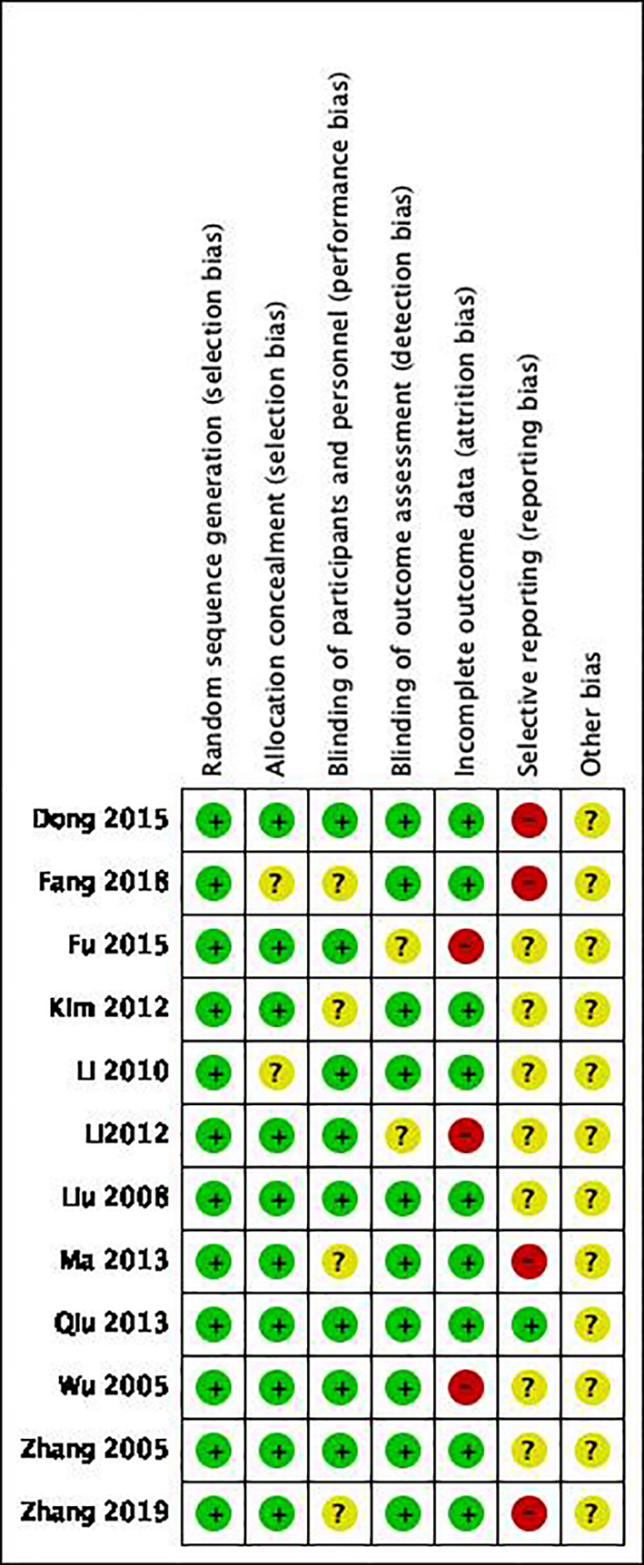
The evaluation results of article quality were included.

A total of 1025 subjects were included in 16 articles from 1998 to 2019. Among them, 478 were treated with HIFU+TACE and 547 were treated with TACE alone. And the quality of the article is evaluated (**Figures 3**, **4**). Among them, 4 articles were included as hepatocellular carcinoma, and the remaining 8 articles were included as primary liver cancer with no pathological types (**Table 1**).

**Table 1:** Basic information of the included literature.

Study + Year	Group	type of lesion	TNMStage	N	Sex(m/f)	Age(year)	Child-Pugh class	Nation	Time of data collection
Liu 2008 (13)	HIFU+TACE	HCC	–	43	53/25*	56.0±10.2	A-B	China	2003-2005
	TACE	HCC	–	35		56.0±10.2	A-B		
Qiu 2013 (14)	HIFU+TACE	PLC	III-IV	39	58/18*	29-65	A-C	China	2007-2012
	TACE	PLC	III-IV	37		29-65	A-C		
Zhang 2005 (15)	HIFU+TACE	PLC	III-IV	55	53/2	65.5±11.3	A-C	China	2000-2005
	TACE	PLC	III-IV	50	45/5	62.3±14.5	A-C		
Ma 2013 (16)	HIFU+TACE	PLC	III-IV	35	26/9	35-64	–	China	2007-2008
	TACE	PLC	III-IV	103	66/37	28-72	–		
Dong 2015 (17)	HIFU+TACE	PLC	III-IV	34	30/4	60.5±7.6	A-B	China	2010-2012
	TACE	PLC	III-IV	31	28/3	61.3±9.2	A-B		
Fu 2015 (18)	HIFU+TACE	PLC	III-IV	36	40/36*	30-70	A-B	China	2011-2014
	TACE	PLC	III-IV	40		30-70	A-B		
Fang 2018 (19)	HIFU+TACE	PLC	–	60	42/18	54.0±5.3	A-C	China	2013-2015
	TACE	PLC	–	66	47/19	54.8±5.1	A-C		
Wu 2005 (20)	HIFU+TACE	HCC	IV	24	15/9	44.5±8.4	A-B	China	1998-2000
	TACE	HCC	IV	26	21/5	47.0±12.6	A		
Li 2010 (21)	HIFU+TACE	HCC	II-IV	44	36/8	29–75	A-B	China	2001-2004
	TACE	HCC	II-IV	45	35/10	30–69	A-B		
Kim 2012 (22)	HIFU+TACE	HCC	I-II	25	18/7	56.0±7.2	A-B	Korea	2006-2009
	TACE	HCC	I-II	32	23/9	65.0±10.0	A-B		
Zhang 2019 (23)	HIFU+TACE	PLC	III-IV	50	25/25	56.0±11.0	A-C	China	2015-2018
	TACE	PLC	III-IV	50	26/24	55.0±10.0	A-C		
Li 2012 (24)	HIFU+TACE	PLC	II-IV	33	23/10	31-77	A-B	China	2006-2010
	TACE	PLC	II-IV	32	24/8	28-69	A-B		

### Tumor response rate

**Figure 5:**
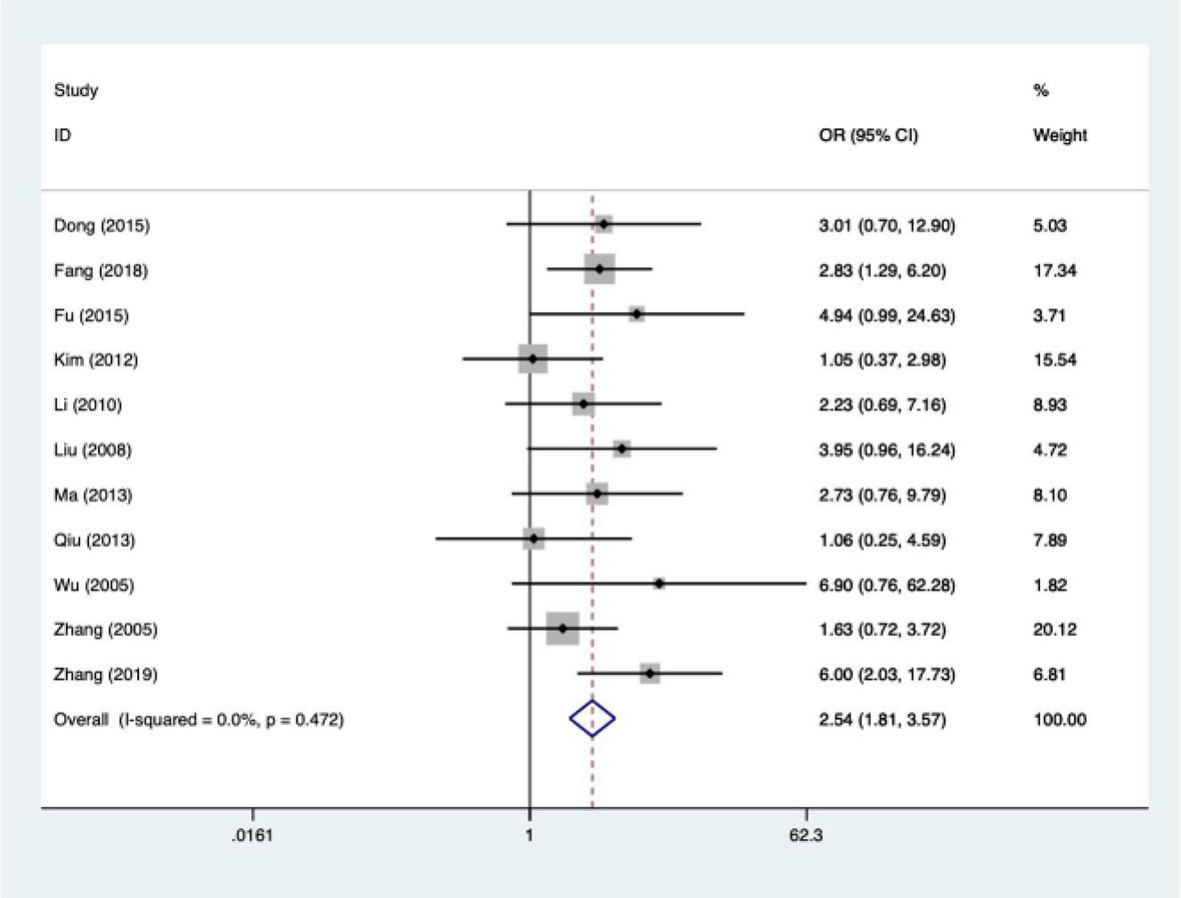
Results of heterogeneity analysis of tumor response rate.

**Figure 6:**
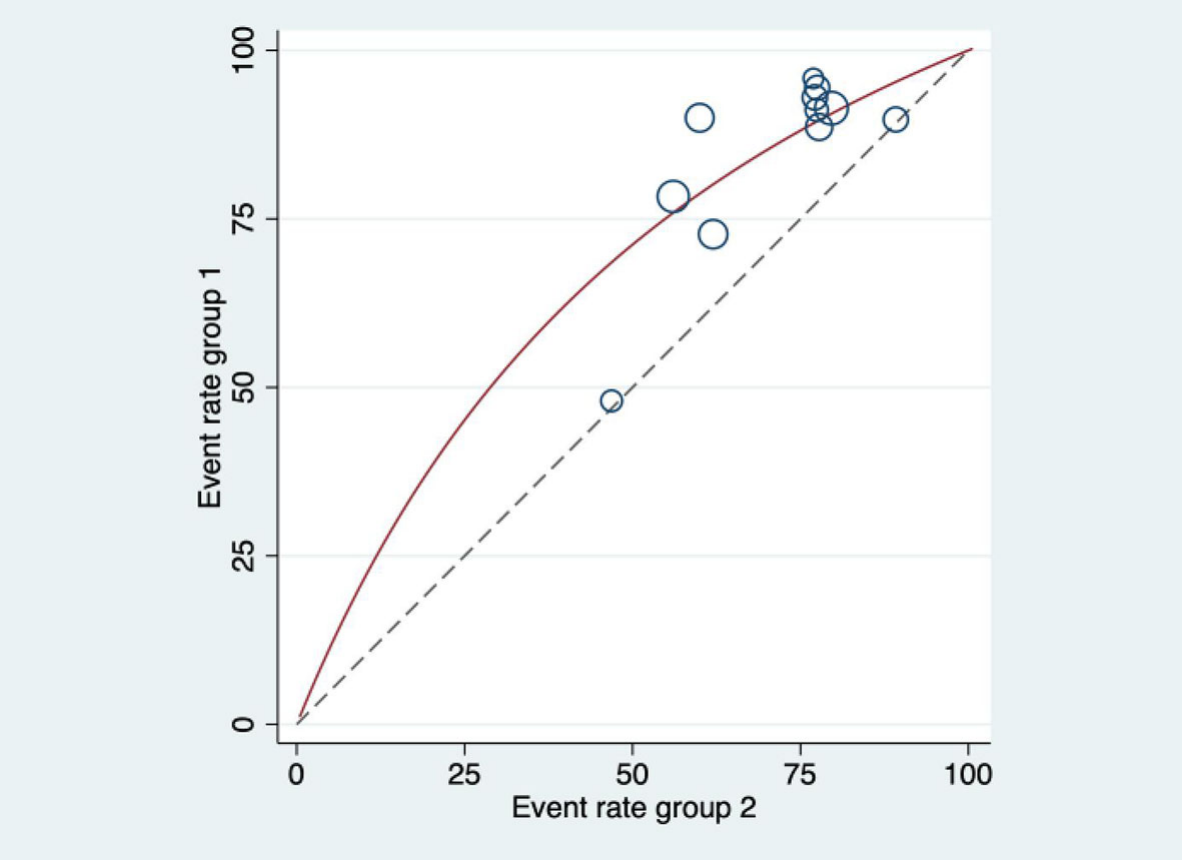
Labbe diagram for heterogeneity evaluation. The dotted line in the figure is the effective line, and the red curve is the combined value. The distance between the two lines represents the magnitude of heterogeneity.

Among the 10 included literatures, the efficacy test results suggested that HIFU combined with TACE had better tumor response efficiency than TACE alone. OR was 2.54 (95%CI: 1.81-3.57). I^2^ = 0.0%, *P*_heterogeneity_ = 0.47. There was no significant heterogeneity among the 11 included articles (**Figure 5**). The efficacy of HIFU combined with TACE in the treatment of liver cancer was 1.54 times higher than that of TACE alone. The heterogeneity was evaluated by drawing Labbe plots (**Figure 6**). Subjective judgment of the graph showed that there was little difference in heterogeneity (the dotted line was the effective line and the red curve was the combined value). The distance between the two curves was small, so the heterogeneity was small. The circles in the figure represent the studies that were included. The image shows dense distribution between studies, which better represents the validity of the paper. One circle was further away, suggesting that the data would be more representative when it was removed in subsequent studies.

### Effect of HIFU on survival rate

#### 6-month survival rate

Heterogeneity test was performed on the 6 included references, and the results showed that *P*_heterogeneity_=0.084, I^2^ = 48.6%, and the 6-month survival rate OR was 4.839 (95%CI:2.889, 8.106). This indicated that there was no significant heterogeneity in 0.5 year survival rate among the 6 articles, and the six-month survival rate of HIFU combined with TACE was 3.839 times higher than that of TACE alone. There was statistical significance between HIFU combined with TACE group and TACE alone group (*P*_significance_<0.05) (**Table 2**).

**Table 2:** Survival rate analysis results.

survival rate	Study	OR	95%CI	I^2^	P_heterogeneity_
Six-months	(13, 14, 17, 18, 20, 21)	4.839	(2.889, 8.106)	48.6%	0.084
one-year	(13, 15–23)	3.306	(2.422, 4.512)	67.4%	0.001
Two-year	(13, 18, 19, 21–24)	2.754	(1.884, 4.025)	0.0%	0.802
Three-year	(13, 15, 16, 18, 21–23)	4.284	(2.783, 6.593)	46.1%	0.084
Five-year	(15, 21–23)	2.367	(1.372, 4.081)	0.0%	0.549

#### 1-year survival rate

Heterogeneity test was performed on 10 included references, and the results showed that *P*_heterogeneity_=0.001, I^2^ = 67.4%, and the One-year survival rate OR was 3.306 (95%CI: 2.422, 4.512). The results indicated that there was significant heterogeneity in one-year survival rate among the 10 articles. There was statistical significance between HIFU combined with TACE group and TACE alone group (*P*_significance_<0.05).

#### 2-year survival rate

Heterogeneity test was conducted for the 7 included references, and the results showed that *P*_heterogeneity_=0.802, I^2^ = 0.0%, and the 2-year survival rate OR was 2.754 (95%CI: 1.884-4.025), indicating that there was no significant heterogeneity in the 2-year survival rate among the 7 articles. The 2-year survival rate of HIFU combined with TACE was 1.754 times higher than that of TACE alone. There was statistical significance between HIFU combined with TACE group and TACE alone group (*P*_significance_<0.05).

#### 3-year survival rate

Heterogeneity test was conducted for the 7 included references, and the results showed that *P*_heterogeneity_=0.084, I^2^ = 0%, and the 3-year survival rate OR was 4.284 (95%CI: 2.783-6.593), indicating that there was no significant heterogeneity in the 3-year survival rate among the 8 articles, and the 3-year survival rate of HIFU combined with TACE was 3.284 times higher than that of TACE alone. There was statistical significance between HIFU combined with TACE group and TACE alone group (*P*_significance_<0.05).

#### 5-year survival rate

Heterogeneity test was conducted for the four included articles, and the results showed that *P*_heterogeneity_=0.549, I^2^ = 0%, and the 5-year survival rate OR was 2.367 (95%CI: 1.372, 4.081), indicating that there was significant heterogeneity in the five-year survival rate among the four articles. There was statistical significance between HIFU combined with TACE group and TACE alone group (*P*_significance_=0.002).

### Heterogeneity analysis of assay indexes

Heterogeneity analysis of AFP, CD3+ and CD4+ showed that *P*_heterogeneity_<0.001 and I^2^>50% (**Table 3**)

**Table 3:** Results of heterogeneity analysis of laboratory indicators.

	Study	WMD	95%CI	Weight%	*P*_heterogeneity_	I^2^	*P*_significance_
AFP	Fu 2015	-300.0	-325.5,-275.4	0.17	<0.001	99%	<0.001
	Liu 2008	-300.0	-325.7,-274.3	0.15			
	Qiu 2013	-93.7	-141.5,-45.9	0.04			
	Zhang 2019	-32.0	-33.0,-31.0	99.6			
CD3+	Dong 2015	12.3	10.11,14.49	48.14	0.001	91%	<0.001
	Fang 2018	7.0	0.76,1.51	51.86			
CD4+	Dong 2015	9.9	7.75,12.05	34.80	<0.001	92%	<0.001
	Fang 2018	5.0	3.43,6.58	65.20			

### Heterogeneity analysis of adverse reactions

Heterogeneity analysis was made for liver dysfunction, postoperative gastrointestinal bleeding, and postoperative pain, and the results showed that there was no significant heterogeneity among the included liver dysfunction studies (*P*_heterogeneity_=0.37, I^2^ = 6%). The incidence of postoperative liver insufficiency was 0.51 times lower in HIFU+TACE group than in TACE alone group. There was significant heterogeneity in gastrointestinal bleeding and postoperative pain (*P*_heterogeneity_<0.001, I^2^>50%) (**Table 4**).

**Table 4:** Results of heterogeneity analysis of adverse reactions.

Adverse reaction	Study	OR	95%CI	Overall OR (95%CI)	*P*_heterogeneity_	I^2^	*P*_significance_
Hepatic Failure	Fang 2018	0.36	(0.04,3.52)	0.49 (0.27,0.89)	0.37	6%	0.01
	Liu 2008	0.52	(0.20,1.33)				
	Qiu 2013	0.82	(0.31,2.14)				
	Zhang 2019	0.11	(0.01,0.89)				
Hemorrhage of digestive tract	Liu 2018	3.56	(1.15,11.02)	1.25 (0.65,2.39)	0.03	72%	0.50
	Qiu 2013	1.11	(0.36,3.43)				
	Zhang 2019	0.25	(0.05,1.25)				
Postoperation pain	Fang 2018	1.11	(0.26,4.64)	0.40 (0.21,0.76)	0.006	80%	0.004
	Li 2010	0.13	(0.05,0.36)				
	Qiu 2013	1.67	(0.37,7.53)				

### Subgroup analysis

The included studies were subgroup analyzed for reasons of heterogeneity in one-year survival.

#### Different pathological types

**Figure 7:**
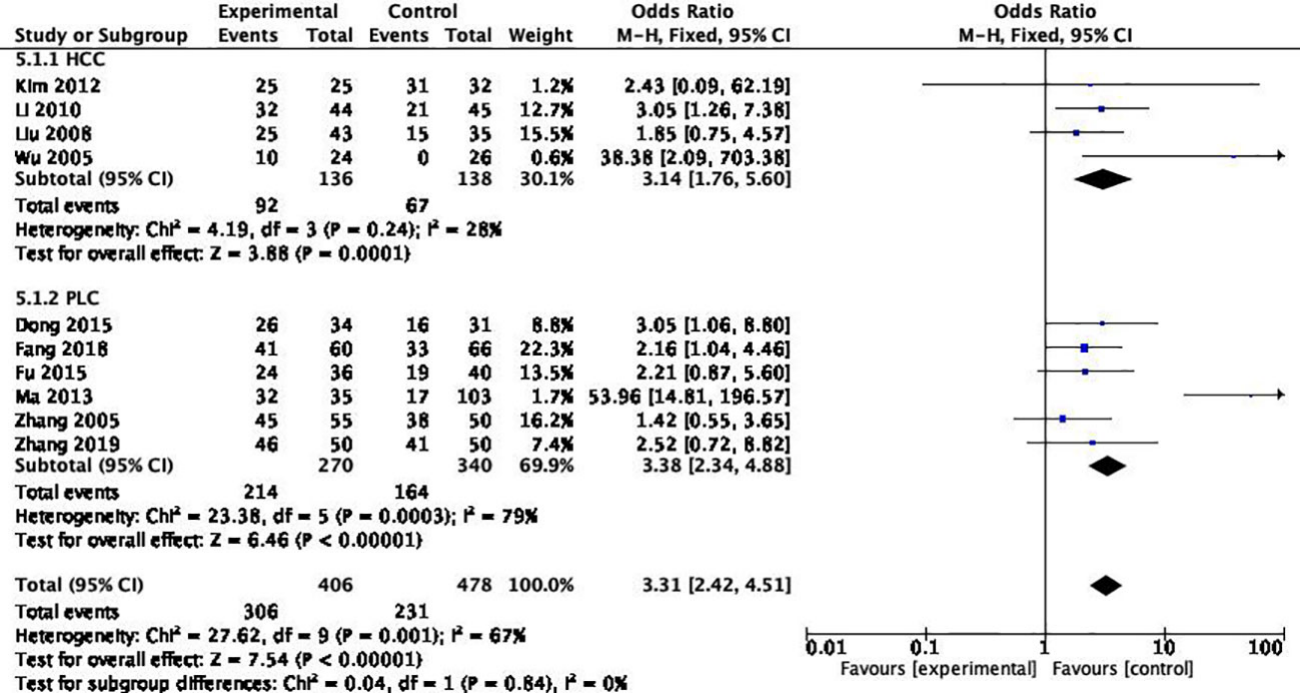
Subgroup analysis of restricted pathologic types.

Among the 10 studies, 4 articles included the pathological type of “hepatocellular carcinoma”, and the remaining 6 articles included “primary hepatocellular carcinoma” without distinguishing the pathological type. The results suggested that there was a high degree of consistency between the PLC groups, suggesting that the differentiation of pathological types was the source of heterogeneity (*P*_heterogeneity_=0.84) (**Figure 7**).

#### Different sources of region

**Figure 8:**
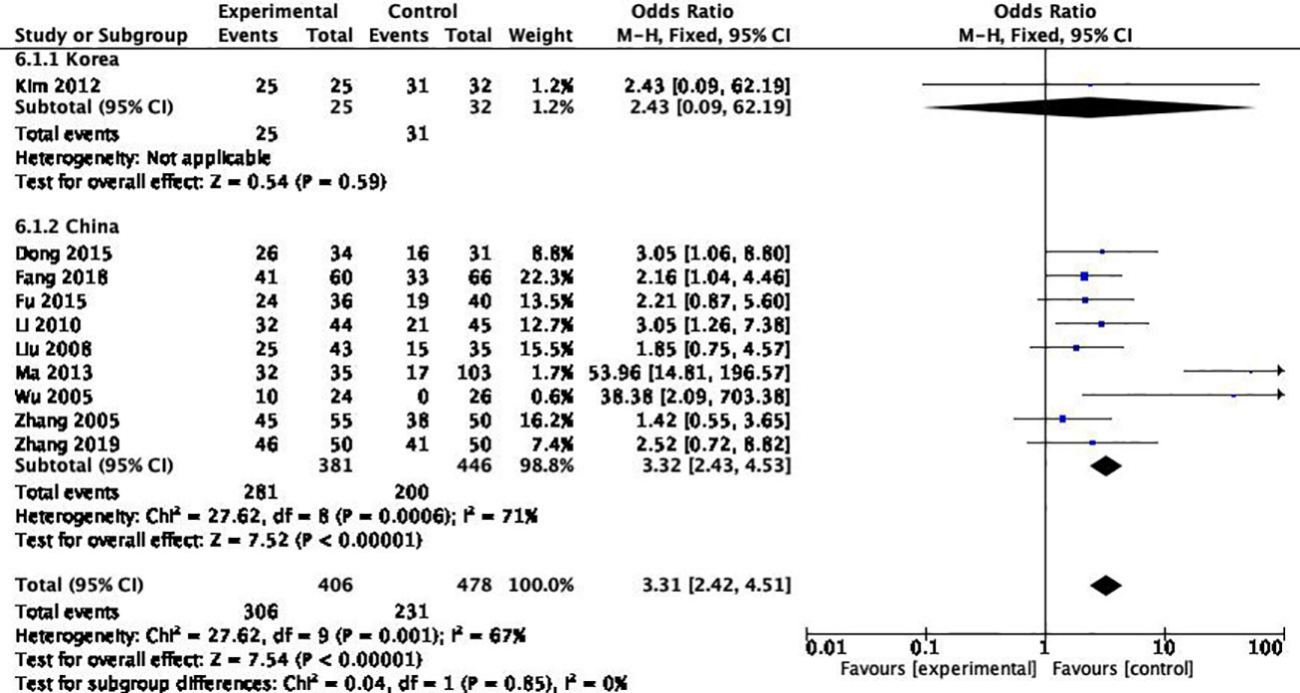
Subgroup analysis of literature sources.

Among the 10 articles, 9 are from China and one is from South Korea. After heterogeneity test, it was found that the source of literature was the source of heterogeneity (*P*_heterogeneity_=0.85) (**Figure 8**).

### Publication bias test (11 articles included)

**Figure 9:**
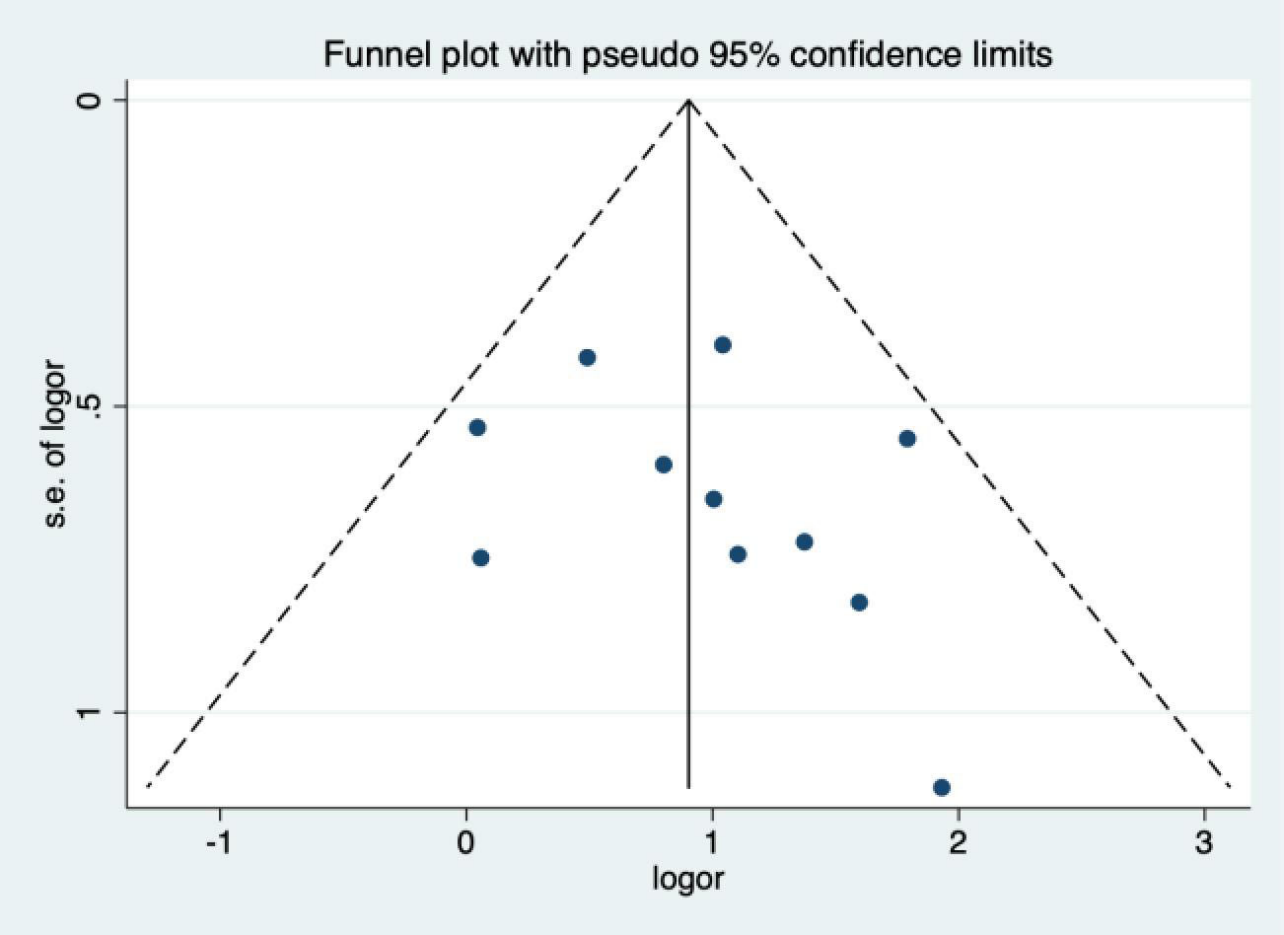
Funnel figure (The symmetry of the black spots in the image reflects the presence of bias).

**Figure 10:**
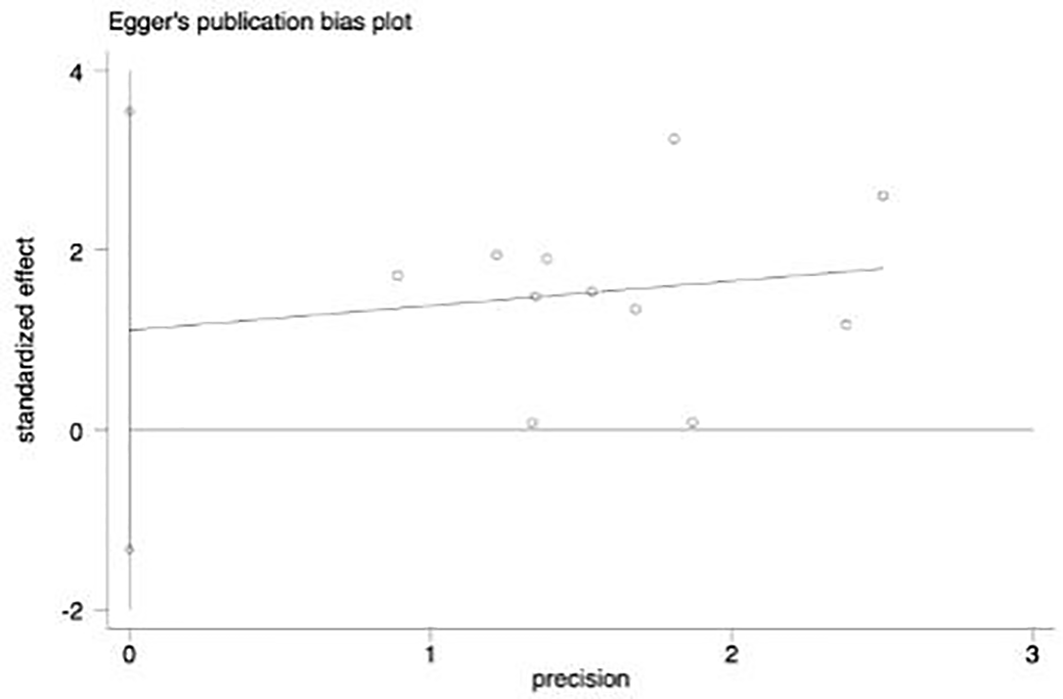
Egger figure. The circles in the figure represent the included articles. It is found in the figure that the included articles are approximately lineardistribution, so there is no deviation.

**Figure 11:**
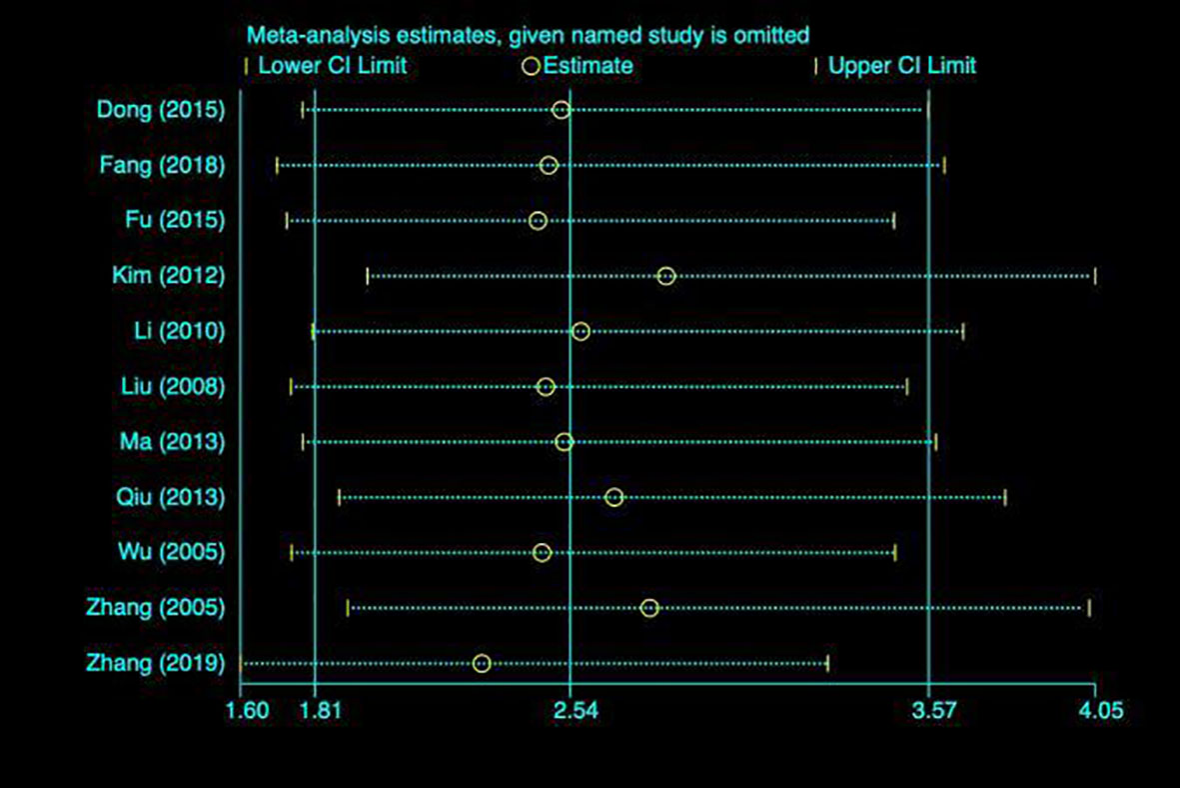
Sensitivity analysis. Each line represents the change in the result after removing the study, and the circle represents the combined effect size after removing the study.

By drawing funnel plot, it can be judged that both sides are almost symmetrical (**Figure 9**). After Egger quantitative analysis(**Figure 10**), 95%CI(-1.333, 3.552), *P*_publication_=0.276. The above quantitative description has no publication bias. Sensitivity analysis was conducted on the impact of single study on the results (**Figure 11**). Each line represents the change in the result after removing the study, and the circle represents the combined effect size after removing the study. The circles were all distributed within 95% confidence interval, indicating that these studies had no significant difference in the results.

### TSA (Sequential trial analysis)

#### TSA was performed for tumor response efficiency

**Figure 12:**
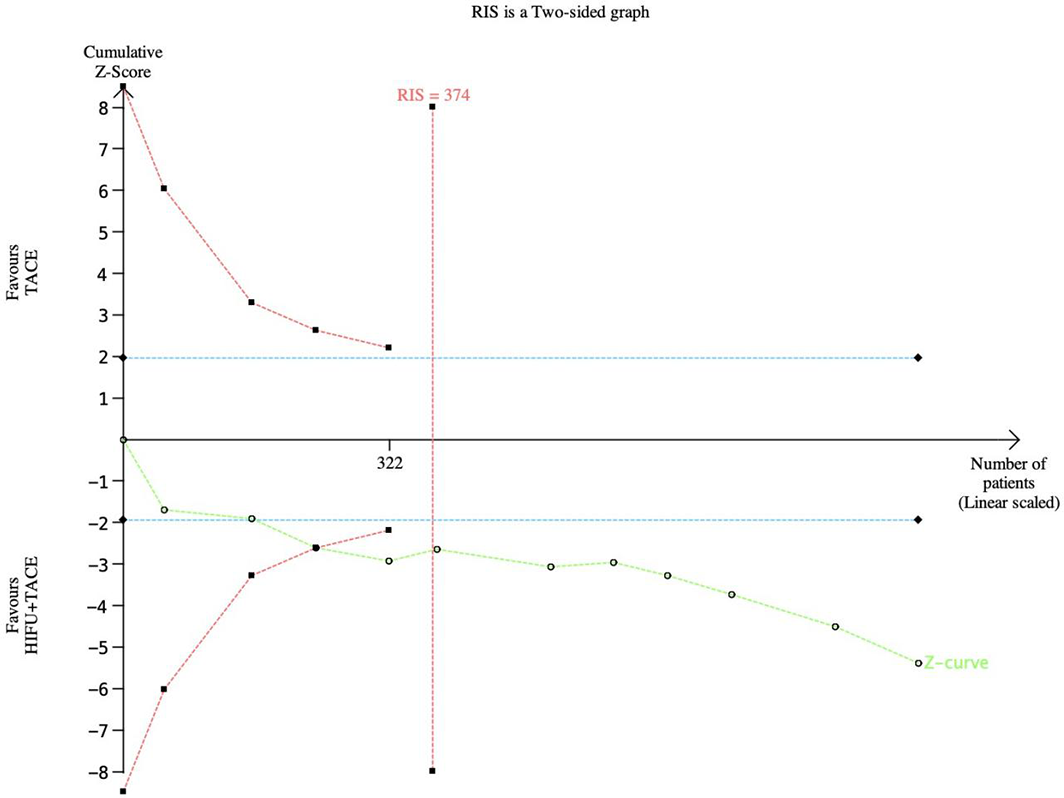
TSA graph of tumor response rate. The pink line represents RIS, the blue line represents the traditional bound, and the green line represents the Z curve.

TSA was performed for tumor response efficiency (**Figure 12**). In this case, the type I error probability α is set to 5%, and the power is set to 80%. The positive rate of the control group was set as 70%, and the relative risk reduction was 20%. In the figure, the blue line represents the traditional boundary (P<0.05, Z=1.96), the red curve represents the TSA boundary, and the green curve represents the Z-curve. As shown in the figure, 374 subjects would have to be enrolled to achieve this desired outcome. In the fourth study, the Z-curve intersected the TSA cut-off value and exceeded the conventional cut-off value. This indicates that the results of meta-analysis are robust.

#### TSA for one-year survival rate

**Figure 13:**
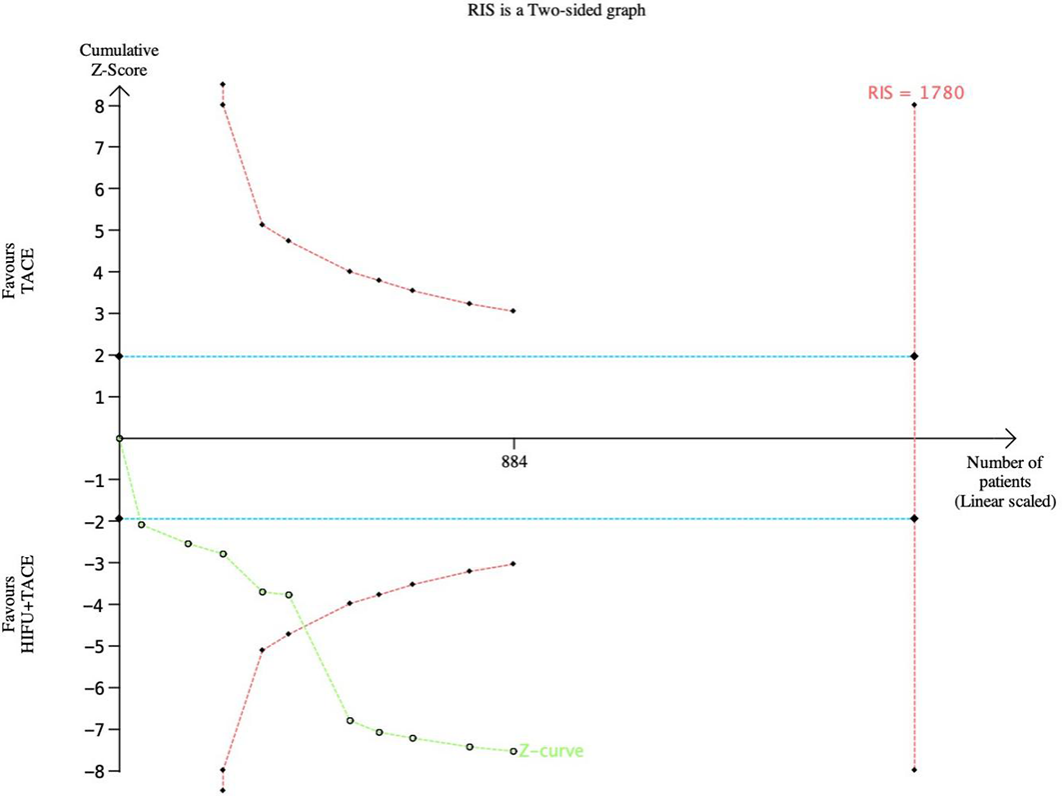
TSA gragh of one-year survival rate. The pink line represents RIS, the blue line represents the traditional bound, and the green line represents the Z curve.

TSA was performed for one-year survival (**Figure 13**). In this case, the type I error probability α is set to 5%, and the Power is set to 80%. The positive rate of the control group was set as 50%, and the relative risk reduction was 25%. In the figure, the blue line represents the traditional boundary (P<0.05, Z=1.96), the red curve represents the TSA boundary, and the green curve represents the Z-curve. As the figure shows, 1780 subjects would have to be enrolled to achieve the expected results. At this time, although the Z-curve did not intersect with RIS, in the sixth study, the Z-curve intersected with the TSA cut-off value, indicating that the meta-analysis results were robust.

### Grade evaluation results

**Figure 14:**
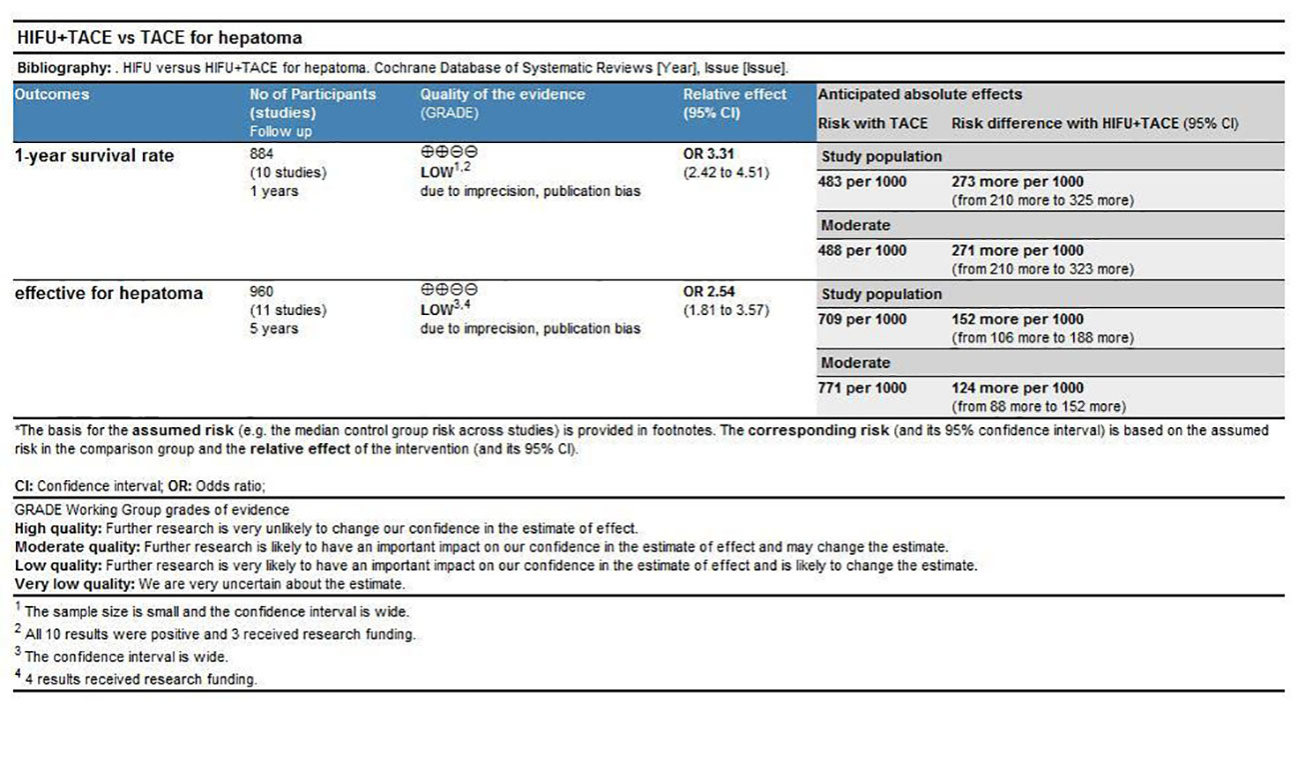
Grade evaluation results.

The results indicated that the recommendation strength of the two indexes(Response rate and one-year survival rate) was low according to the meta-analysis results (**Figure 14**).

## Discussion

Clinically, TACE has achieved good results in the treatment of unresectable liver cancers (25, 26). However, the tumor recurrence rate after TACE is high, causing an increased risk of postoperative liver failure. Therefore, combined therapy is commonly used in clinical practice, including microwave ablation (MWA) (27) and radiofrequency ablation (RFA) (28). Studies have shown that the effect of combined therapy is better than that of single therapy. In this paper, 12 articles were analyzed. The sample size of the included articles was large, and the time span was large. The sensitivity analysis results suggested that the articles were more stable, so the results of the effective rate in this study were more reliable. The overall response rate of tumor response in the HIFU combined with TACE treatment group was significantly better than that in the TACE alone group. With its advantages of precise localization, repeated treatment, and high-intensity thermal ablation, HIFU allows precise coagulative necrosis of tumor tissue and improves the tumor elimination efficiency by 1.54 times.

The results of meta-analysis showed that there were significant differences in the half-year, one-year, two-year, three-year and five-year survival rates between the combined treatment group and the TACE alone group, and the survival rate was improved by 1-4 times compared with the TACE alone group. In TACE, the recurrence rate of tumors will be increased due to hepatic artery obstruction and compensation of the system (29). Therefore, after TACE, HIFU is consistent with the principle of enhanced energy absorption after contrast agent injection (30, 31), enhancing the thermal effect on the tumor and more conducive to its elimination, thus effectively prolonging the survival time of patients.

Meta-results of AFP, CD4+, and CD3+ indicated that postoperative differences between the combined treatment group and the single treatment group were statistically significant, indicating that the combined treatment was better than TACE alone. Meta-analysis of adverse reactions showed that there were statistically significant differences in postoperative pain and postoperative liver insufficiency between the two groups, and the risk of the two adverse reactions in the combined treatment group was lower than that in the single group. However, the difference in gastrointestinal bleeding between the two groups was not statistically significant, indicating that HIFU had little influence on the occurrence of this adverse reaction. The reason for this result may be that HIFU technology can accurately eliminate tumors, relieve the pressure of tumors on surrounding tissues, and reduce pain. Due to the precision of HIFU operation, it has little influence on the surrounding normal liver tissue, and HIFU has the advantage of no damage to the target blood vessels, and the two aspects jointly reduce the probability of postoperative liver insufficiency.

The sources of heterogeneity in one-year survival were analyzed by subgroup. The results indicated that heterogeneity was caused by country, pathological type and study type. After sorting out the included literature, it was found that most of the articles were from China, which may be related to the origin and application scope of HIFU technology.

TSA results indicated that although the Z curve crossed the traditional threshold, it did not cross the TSA term value. The results showed that the sample size was sufficient, but there may be a false positive conclusion. More experiments are still needed to prove whether the tumor response rate and one-year survival rate of HIFU combined with TACE are better than that of TACE alone.

Grade evaluation results indicated that the quality of evidence for response rate and one-year survival was low. Shortcomings of this study:(1) the study endpoints included in this study were limited to survival rate or tumor response rate, which made other articles not included; (2) There was no restriction on the types of articles included, and some articles did not follow the requirements of randomized controlled experiments; (3) TSA results suggested that there were only a few articles included, so the scope of the study needed to be expanded. More literatures from different countries on the efficacy of HIFU combined with TACE could be included in the subsequent study. (4) There might be unknown confounding factors influencing the results.

## Conclusion

The results of meta-analysis and TSA showed that HIFU combined with TACE had better efficacy in tumor elimination and improved survival rate compared with TACE alone. However, more and broader studies need to be included to further confirm the authenticity of this conclusion.

## Data availability statement

The raw data supporting the conclusions of this article will be made available by the authors, without undue reservation.

## Author contributions

Conception and design: LZ and KX. Administrative support: KX, LL. Provision of study materials or patients: JC, XZ and KX. Collection and assembly of data: LZ, XZ and JC. Data analysis and interpretation: LZ and KX. Manuscript writing: all authors. Final approval of manuscript: all authors.

## Conflict of interest

The authors declare that the research was conducted in the absence of any commercial or financial relationships that could be construed as a potential conflict of interest.

## Publisher’s note

All claims expressed in this article are solely those of the authors and do not necessarily represent those of their affiliated organizations, or those of the publisher, the editors and the reviewers. Any product that may be evaluated in this article, or claim that may be made by its manufacturer, is not guaranteed or endorsed by the publisher.
